# Increased Knowledge Mediates the Effect of Game Changers for Cervical Cancer Prevention on Diffusion of Cervical Cancer Screening Advocacy Among Social Network Members in a Pilot Trial

**DOI:** 10.1007/s12529-023-10217-7

**Published:** 2023-09-01

**Authors:** Ishita Ghai, Glenn J. Wagner, Joseph K. B. Matovu, Margrethe Juncker, Eve Namisango, Kathryn Bouskill, Sylvia Nakami, Jolly Beyeza-Kashesya, Emmanuel Luyirika, Rhoda K. Wanyenze

**Affiliations:** 1 Frederick S. Pardee RAND Graduate School, RAND Corporation, Santa Monica, CA USA; 2 RAND Corporation, Santa Monica, CA USA; 3 School of Public Health, Makerere University, Kampala, Uganda; 4 Faculty of Health Sciences, Busitema University, Mbale, Uganda; 5 Rays of Hope Hospice Jinja, Jinja, Uganda; 6 African Palliative Care Association, Kampala, Uganda; 7 Mulago Specialized Women and Neonatal Hospital, Kampala, Uganda; 8 School of Medicine, Makerere University, Kampala, Uganda

**Keywords:** Cervical cancer, Prevention, Peer advocacy, Intervention, Randomized controlled trial, Mediation, Social diffusion, Social networks

## Abstract

**Background:**

Game Changers for Cervical Cancer Prevention (GC-CCP), a peer-led, group advocacy training intervention, increased cervical cancer (CC) prevention advocacy not only among intervention recipients, but also their social network members (referred to as “alters”) who were targeted with advocacy in a pilot randomized controlled trial. We examined mediators and moderators of this effect on alter advocacy, to understand how and for whom the intervention had such an effect.

**Method:**

Forty women (index participants) who had recently screened for CC enrolled and were randomly assigned to receive the GC-CCP intervention (*n* = 20) or the wait-list control (*n* = 20). Up to three alters from each participant (*n* = 103) were surveyed at baseline and month 6. Measures of CC-related cognitive constructs (knowledge, enacted stigma, and risk management self-efficacy), as well as extent of advocacy received from index participants, were assessed as mediators of the intervention effect on alter advocacy using multivariate regression analyses. Alter characteristics were examined as moderators.

**Results:**

Increased CC-related knowledge partially mediated the intervention effect on increased alter engagement in CC prevention advocacy; those with greater gains in knowledge reported greater engagement in advocacy. No moderators of the intervention effect were identified.

**Conclusion:**

The effect of GC-CCP on alter CC prevention advocacy is enhanced by increased alter knowledge pertaining to CC prevention, causes, and treatment and suggests this may be key for diffusion of intervention effects on increased CC prevention advocacy throughout a social network.

**Trial Registration:**

NCT04960748 (registered on clinicaltrials.gov, 7/14/2021).

**Supplementary Information:**

The online version contains supplementary material available at 10.1007/s12529-023-10217-7.

## Introduction

The International Agency for Research on Cancer (IARC) estimates that cervical cancer (CC) is the second most diagnosed cancer among women, with over 600,000 new cases worldwide in 2020. It is concurrently one of the leading causes of mortality among women [1]. Importantly, the incidence of CC in Uganda is among the highest in the world (with an age-standardized incident rate of 56.2 cases per 100,000) [1], and over 20% of all cancer-related deaths to Ugandan women was attributed to CC in 2020 [1]. Vaccinations that guard against human papilloma virus are recommended to be given at an early age [2] but are only just beginning to become available in Uganda [3, 4]. As such, the best way to prevent CC-related mortality is through early detection. Periodic screenings with methods such as visual inspection of the cervix with acetic acid (VIA), along with treatment for pre-cancerous lesions, are largely available free of charge or at a low cost in some areas in Uganda. Moreover, radiation therapy is available for advanced disease but is largely unaffordable and only available at one location.

Despite the importance of early, periodic CC screening, studies have estimated that the proportion of women in Uganda who have ever been screened for CC is between 5 and 30% [5–7], including 8% over the 2014–2019 period [8]. Furthermore, over 80% of women first present for care when they already have advanced disease [7]. The contributing factors to this low uptake of CC screening include structural barriers (lack of trained staff, low availability of screening and treatment services in much of the country), psychological barriers (competing priorities, low knowledge and corresponding fears and misconceptions), and social barriers (stigma associated with screening and treatment) [5, 7, 9, 10]. The Uganda Ministry of Health, through the national Strategic Plan for Cervical Cancer Prevention and Control guideline (2010–2014), is slowly beginning to address the structural barriers, but parallel efforts to address the psychological and social barriers are needed to increase CC screening and disease prevention.

A promising approach to address the social and psychological barriers to CC screening is peer advocacy, wherein those members of the community that have screened for CC risk encourage and empower their peers to also get screened for CC. Peer advocacy has promoted health behavior change in the context of HIV [11–14] but has not been studied in the context of CC prevention, despite evidence suggesting that encouragement from others to get screened and knowing someone who has screened for or been diagnosed with CC are facilitators of CC screening uptake [15].

**Fig. 1:**
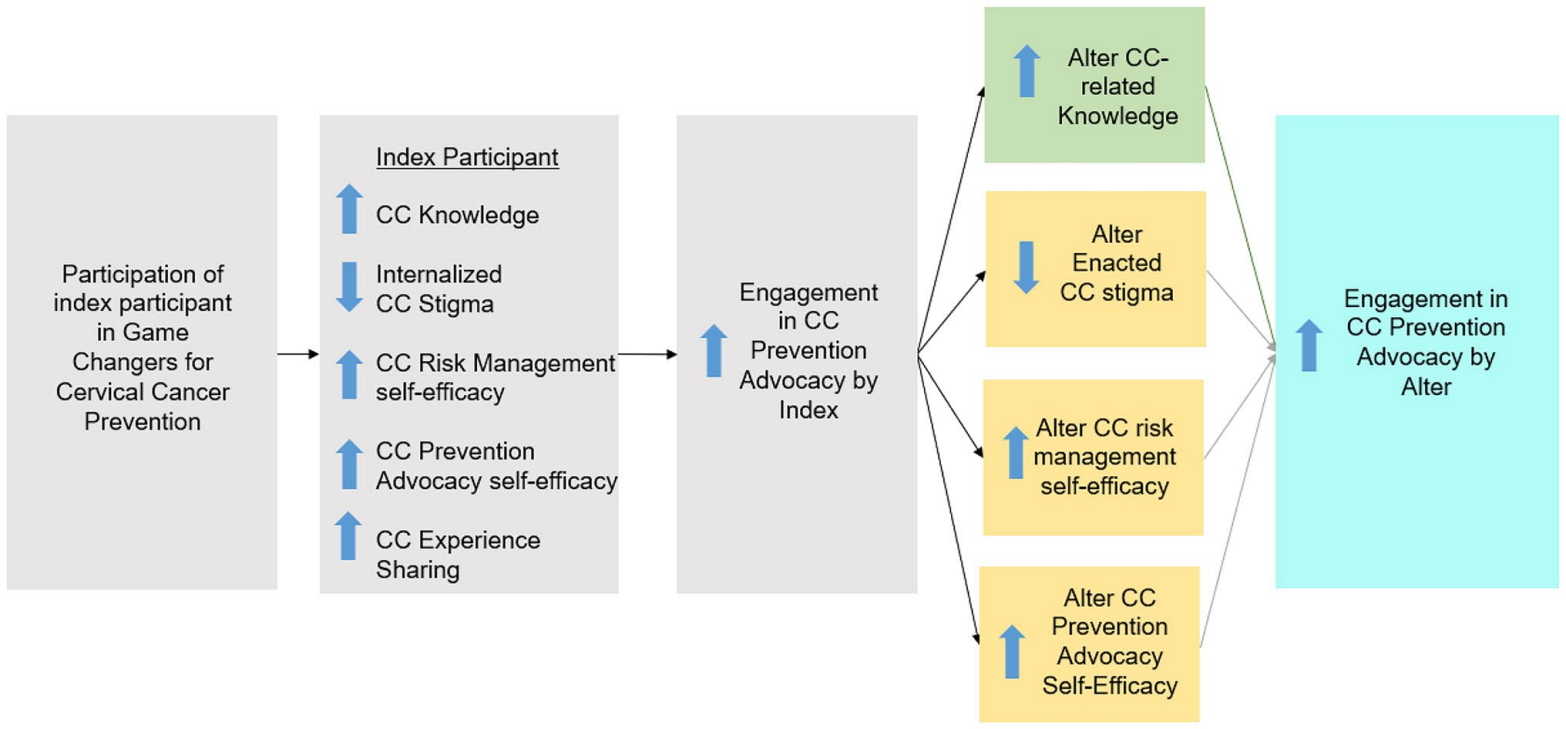
Conceptual framework for the mediators of the downstream effect of Gamer Changers for Cervical Cancer Prevention (GC-CCP) intervention on cervical cancer (CC) prevention advocacy by social network members (alters)

We developed a social network-based, group advocacy training intervention — Game Changers for Cervical Cancer Prevention (GC-CCP) — to promote CC screening by empowering women who had recently screened for CC risk to engage in CC prevention advocacy among women in their social networks. GC-CCP draws on theories of social diffusion [16], cognitive consistency [17], and social influence [18], which posit that behavior change can be initiated by a few and diffused to others through modeling, advocacy, and shifts in social norms. The conceptual framework for the intervention posits that gains in CC knowledge, comfort sharing one’s CC screening experience, and skills and self-efficacy related to CC risk management and CC prevention advocacy, as well as reduced internalized CC stigma, will increase engagement in CC prevention advocacy among intervention recipients and that the targets of that advocacy would also increase their own CC prevention advocacy as a result of similar cognitive processes (see Fig. 1).

A pilot randomized controlled trial (pilot RCT) of the GC-CCP intervention revealed a dramatic effect on alter CC screening with over half of previously unscreened alters of women in the intervention group being screened for CC during the 6-month study period, compared to less than one seventh of those in the control group [19]. The intervention also showed an effect on its most direct target, increased CC prevention advocacy among intervention recipients in the pilot results. Furthermore, an intervention effect was also found on increased alter engagement in CC prevention advocacy [19]. This diffusion of advocacy effect is in line with the overall intent of network-based interventions, whereby information and behavior change are diffused quickly and efficiently through connected peers within a network — not only to the immediate peers of a particular network node (i.e., a person), but also their second and third degree connections [20–22]. To the extent that this diffusion effect can be maximized, the GC-CCP intervention can have a greater impact on CC screening uptake and prevention within networks and the larger community.

To identify the mechanisms that drive the intervention effect on increased alter advocacy in the pilot trial referenced above, we examined whether cognitive constructs in our conceptual framework (as detailed in Fig. 1) acted as mediators of the intervention effect on increased engagement in CC prevention advocacy by alters. We also explored alter characteristics that may moderate the effect of the intervention, to better understand the context in which the intervention may be particularly effective in increasing alter CC prevention advocacy.

## Methods

### Study Design

**Fig. 2:**
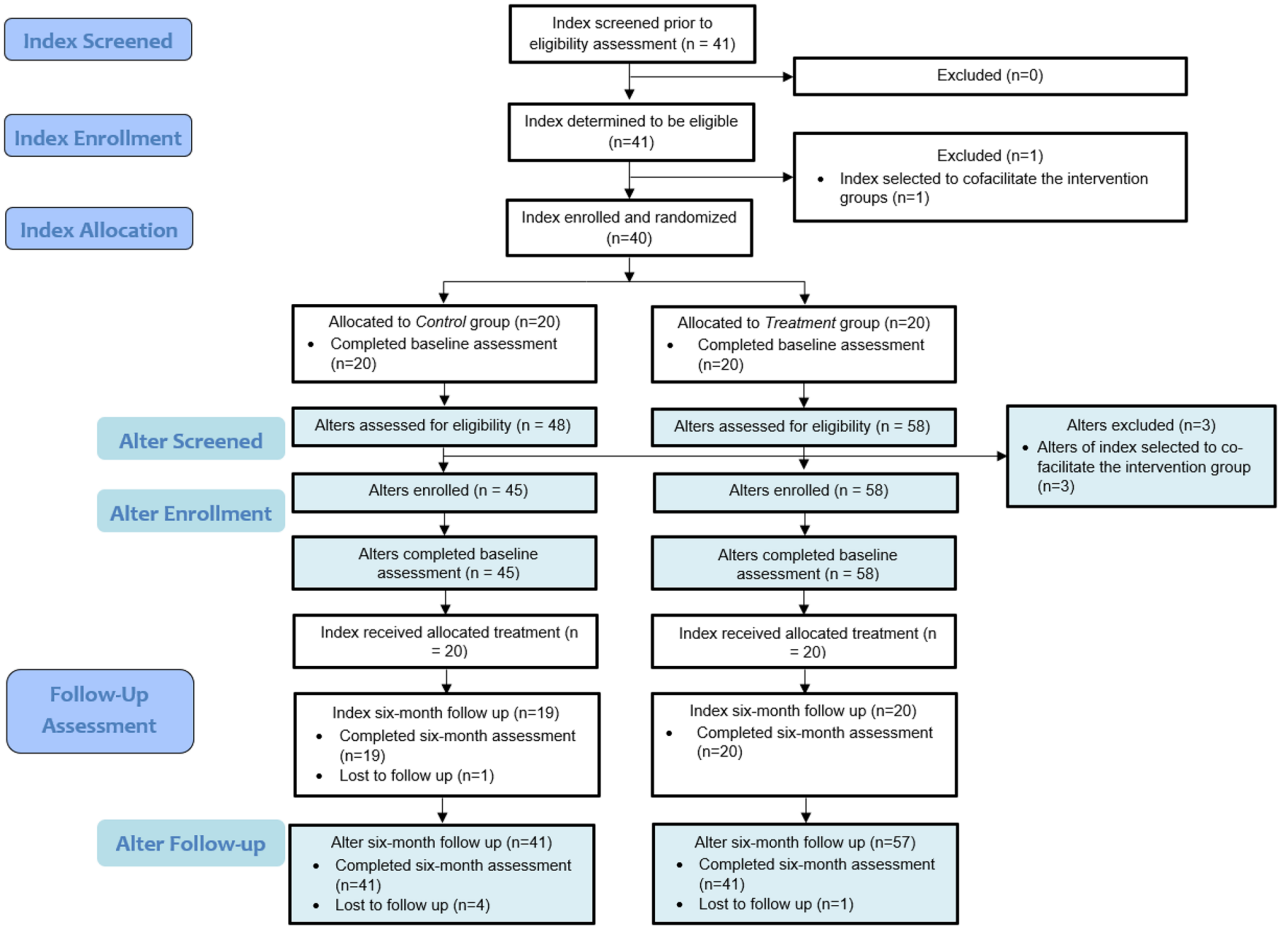
Adapted CONSORT flow diagram for GC-CCP intervention

The study was a pilot randomized controlled trial of Game Changers for Cervical Cancer Prevention (GC-CCP). Forty women who had screened for CC within the past year (referred to as index participants) were randomized to receive the GC-CCP intervention (*n* = 20) or the wait-list control group (*n* = 20), with individual randomization on a 1:1 ratio (please see the CONSORT diagram in Fig. 2 [23]). Randomization was carried out using a random number generator. Women randomized to the wait-list control group received the intervention after data collection was completed. Randomization was stratified by age (under and over age 35) and history of CC-related treatment. Participants were not blinded to assignment due to the nature of the intervention; only the data analyst was blinded. Since this was a pilot trial with limited funding resources, a sample size was chosen that would suffice for evaluation of feasibility and acceptability, rather than efficacy.

Each index participant was asked by the study coordinator to enroll up to three women in their social network (referred to here as “alters”) who had not screened for CC in the past three years (though all alters reported never having been screened). All participants (index and alter) were administered assessments at baseline and month 6 and received 30,000 Uganda shillings (~ $8 USD) for each completed assessment; however, only data from the alter participants was included in the analysis reported in this paper. The study protocol was reviewed and approved by the Makerere University School of Public Health Research and Ethics Committee, and the Uganda National Council for Science and Technology, and registered on ClinicalTrials.gov (NCT04960748) on July 14, 2021.

### Study Setting

The study was conducted between September 2021 and April 2022 at Buyinja Health Center IV, in Namayingo district, a rural community in the Busoga region of Uganda. CC screening and thermal therapy for pre-cancerous lesions are available at the site, as well as at a nearby health center (Banda Health Center III), and through Rays of Hope Hospice Jinja (RHHJ), which conducts periodic mobile CC screening and thermal therapy “day camps.” Women who need biopsies are sent to Jinja (approximately 90 km from Namayingo), and if cancerous lesions are present, they are referred to the Uganda Cancer Institute in Kampala (approximately 200 km from Namayingo). RHHJ tracks women who are screened for CC as part of their camps, to facilitate follow-up care.

### Participants

Index participants were enrolled in the study if they were aged 18 years or older, had screened for CC within the past year, had stable health status (i.e., not in end stages of disease, so that they were likely to complete the 6-month study follow-up), and had shared their CC screening experience with at least one woman who they perceived to not have screened for CC in the past three years. Alter participants were eligible if they were at least 18 years of age, were recruited by a woman who was enrolled as an index participant, and self-reported not being screened for CC in the past 3 years. All participants had to speak either Samia or Lusoga, the two prominent languages in Namayingo district.

Recruitment took place in September 2021. Index participants were recruited through the RHHJ database of women who had received CC screening within the past 12 months and referrals from Buyinja and Banda health center providers. This recruitment was purposive to recruit a balance of women who screened positive for signs of CC risk (pre-cancerous or cancerous lesions) and women who screened negative. An RHHJ staff member or health center provider informed eligible women of the study and those who expressed interest were referred to the study coordinator for formal eligibility screening and consent procedures. After providing written informed consent, women were administered the baseline assessment and then randomly assigned to the intervention or control arm.

Each index participant was asked by the study coordinator to enroll up to three alters in their social network who had not screened for CC in the past 3 years, but with whom the index participant had shared her CC screening experience. Recruiting alters to participate in a study related to CC could lead the alter to assume that the index participant has been screened for CC or even has CC. Thus, recruiting alters who already knew the index participant’s CC screening experience helped avoid any risks associated with unintentional disclosure (such as stigma). The baseline survey for index participants included listing up to twelve women in their social network and describing their characteristics. To recruit alters, we randomly selected five alters who the index participant reported as knowing her CC screening experience (or as many as there were if less than five) and who were believed by the index not to have been screened for CC; the index participant was then asked if she was comfortable asking three of these alters to participate. The index participant was asked to call each selected alter at the end of the interview to describe the study in the presence of the coordinator, who then scheduled a study visit if the alter expressed interest in participating. If an alter refused or could not be reached, a replacement was randomly selected from the list of alters who knew the index participant’s CC screening experience (and whom the index participant was comfortable recruiting). When screening the alter, the coordinator confirmed that the individual was not already recruited by another index. All participants provided written informed consent.

### Intervention

The intervention consisted of seven weekly group sessions. *Session 1* focused on addressing fears and concerns related to CC risk and use of self-compassion and peer support to overcome fears and internalized stigma, as well as introducing the overall vision for empowering women to become change agents for CC prevention and treatment. *Session 2* focused on building skills and decision-making for sharing one’s personal CC screening experience, knowing to whom to disclose and when, and how to initiate and navigate disclosure and conversations about CC. *Session 3* built skills and motivation for recognizing signs of CC risk and seeking health services, so that the advocate’s own behavior was consistent with the behavior they encouraged in others, as well as instruction on facts and myths related to CC to facilitate accurate CC screening advocacy. *Session 4* introduced the concept of a social network and how one’s network can serve as a tool for CC prevention advocacy and dissemination of CC-related information. *Sessions 5 and 6* focused on the skills needed for successful CC prevention advocacy, including strategies for how to start and sustain conversations about CC, and effective communication skills (e.g., reflective listening, paraphrasing, and open-ended questions). *Session 7* focused on peer solidarity and support to inspire a commitment to ongoing CC advocacy. The sessions were administered in a group format to facilitate sharing of experiences to build support, solidarity, and motivation among participants; group problem solving and role playing to build skills and self-efficacy; setting personal goals regarding disclosure and advocacy; and the use of take-home activities to reinforce practice of new skills and generate personal experiences to be processed in the sessions. Each session lasted 120–150 min. Participants received 30,000 Uganda shillings (~ $8 USD) for attending each session to cover transport costs.

The sessions were conducted using a structured facilitator manual, in the predominant local languages of Samia and Lusoga, by two women facilitators from Namayingo who had ever screened for CC. The facilitators were trained by the senior investigators over 3 days. The supervisor of the facilitators observed the implementation of each session and provided feedback and further training as needed during weekly supervision. More information on the intervention and the study design can be found elsewhere [19].

### Measures

The survey was administered in either Samia, Lusoga, or English (depending on the preference of the participant) using Network Canvas [24], a computer-assisted software. Measures were translated using standard translation/backtranslation methodology [25]. All measures were developed by the study team, except those in which an attribution is cited. For measures developed by the study team that included at least three items, we cite internal reliability statistics (Cronbach’s alpha) [26], except in instances where the items do not lend themselves to a scale (such as the measure for *Receipt of CC screening advocacy*).

### CC Prevention Advocacy

CC advocacy prevention was measured using a six-item scale that asks respondents whether they had discussed specific topics pertaining to CC with people they know, on a scale of 1 (not at all) to 5 (very much). Topics included importance of getting screened for risk of CC, how and where to get screened, how to prevent CC, the need to use condoms to limit the risk of getting HPV-infected, benefits of having fewer sexual partners in limiting the risk of HPV infection and subsequent CC risk, and the importance of getting treatment if CC risk is suspected. The mean of items was calculated; higher mean indicated higher engagement in CC prevention advocacy (Cronbach’s alpha = 0.89).

### Potential Mediators

Several variables in the alter assessment were selected as potential mediators based on our conceptual framework for factors that may influence alter engagement in CC prevention advocacy (see Fig. 1). *CC-related knowledge* was assessed with 16 statements or questions reflecting the etiology, prevention, and treatment of CC (see Supplementary File 2 for details). A sum of correct responses was calculated for analysis (Cronbach’s alpha = 0.70). *CC enacted stigma* was assessed through six items adapted from measures developed by Marlow and Wardle [27] and Cho et al. [28]. Participants were asked to rate their agreement with statements (e.g., a woman with cervical cancer is to blame for her condition; I feel uncomfortable when I am around women with cervical cancer) by indicating they 1 “disagree”; 2 “I neither agree nor disagree. I do not have a feeling either way”; or 3 “agree.” Mean item scores were calculated, with higher scores reflecting greater stigma (Cronbach’s alpha = 0.40). *CC risk management self-efficacy* was assessed with three items that measured confidence to notice a symptom of CC risk, seek health services for a symptom of CC risk, and obtain treatment if screening revealed signs of CC risk. For each item, the level of confidence was reported using response options ranging from 0 “you cannot do it at all” to 10 “you are completely certain you can do it”; the mean item score was calculated (Cronbach’s alpha = 0.66). *Receipt of CC screening advocacy* from the index participant who referred them to the study was assessed with a series of four questions. The participant was first asked (1) if the index participant had talked with them about getting screened for CC in the past 6 months, and if the response was “yes,” the participant was asked if this discussion included the index participant (2) encouraging the respondent to get CC screened; (3) providing information about where to get screened; and (4) providing direct support to the respondent to get screened. For each question, the response option was 1 “yes” or 0 “no”; the mean of responses for items 2–4 was calculated.

### Potential Moderators

These alter variables included age (whether the participant was over 35 years old), education (whether the participant had received any secondary education), and relationship status (whether the participant was in a committed relationship). These demographic variables were selected considering the possibility that the intervention effects could be larger for women who are older, more educated, and have a partner. In addition, social support was measured using four items adapted from the Medical Outcomes Study Social Support Survey [29] that asked participants to indicate how often they had access to specific types of social support (e.g., someone to take them to the doctor; someone to help suggest solutions to problems) on a scale of 1 “none of the time” to 5 “all of the time”; the mean item score was calculated (Cronbach’s alpha = 0.74). This was included as a potential moderator with the hypothesis that those who perceived their social network members to be more supportive would feel more comfortable engaging in advocacy.

### Data Analysis

To examine intervention effects on alter engagement in CC prevention advocacy, we used a multiple linear regression analysis with the month 6 measure of alter advocacy being the dependent variable, while independent variables included the baseline measure of the dependent variable and an indicator of intervention condition (intervention or control), as well as alter demographics as covariates (age < 36 years, any secondary education, presence of a main sex partner). Separate models were run for the advocacy scale mean, as well as each individual item.

The potential mediators were first examined for significant associations with the intervention to establish their potential for consideration as a mediator of the intervention effect on increased alter engagement in CC prevention advocacy. Multiple linear regression models were developed to estimate the intervention effects on each of the potential mediators. In each model, the month 6 measure of the potential mediator was the dependent variable, and the intervention condition (intervention or control group) and the baseline measure of the dependent variable were the independent variables. Alter demographics (whether the alter was older than 35 years, any secondary education, relationship status) were included as covariates. An intent-to-treat analysis was carried out, with any missing values at month 6 imputed with the baseline value of the same measure for a conservative estimation. We examined correlations between the potential mediators and the month 6 measure of alter CC prevention advocacy using Pearson correlation coefficients.

To test for mediation, we used a structured equation modeling approach using STATA v17, along with bootstrap resampling, to compute standard errors and confidence intervals. A two-step approach was used to test for mediation. In step one, the dependent variable was the month 6 measure of CC prevention advocacy, while independent variables were the intervention condition and the baseline measure of the mediator; the three alter demographics remained as covariates. In step two, the month 6 measure of the mediator was added to the model, resulting in the model assessing the mediating effect of change in the mediator from baseline to month 6.

To test for moderation of the intervention effect on CC prevention advocacy, for each potential moderator, we used multiple linear regressions to model CC prevention advocacy at month 6 as the dependent variable. Independent variables included the intervention condition, the moderator measured at baseline, and their interaction. Additionally, we used the *margins* command to calculate the coefficients for the intervention condition indicator and the interaction with the moderator at its mean, one standard deviation above the mean, and one standard deviation below the mean to evaluate the effect of the intervention at low, medium, and high levels of the moderator.

## Results

### Sample Characteristics

Forty women (20 who receive their healthcare from Buyinja Health Center IV, 20 from Banda Health Center III) who had screened for CC within the past year were screened for eligibility. All were found to be eligible and were enrolled into the study with a 1:1 distribution in each group (i.e., 20 in treatment and 20 in wait-list control). Twenty-four index participants (60%) had screened positive for signs of pre-cancerous lesions, and each received treatment (17 received thermal therapy, seven received cryotherapy), while the remaining 16 screened negative for any sign of CC risk. The 20 participants in the intervention arm were divided into two groups of ten to receive the seven-session intervention; 19 (95%) attended all seven sessions.

From the 40 index participants, 103 alter participants (45 from index participants in the control group, 58 from index participants in the intervention group) were enrolled. Among the alter participants, 37.9% were over 35 years of age, 29.1% had any secondary education, 81.6% were in a committed relationship, and 5.8% were HIV-positive. Alters of index participants in the intervention group were more likely to have any secondary education (39.7% vs. 15.6%; *p* = 0.01) and to report being in a committed relationship (89.7% vs. 71.1%; *p* = 0.02), compared to the alters of index participants in the control group. Retention at month 6 was high among alter participants, with 98 (41 control, 57 intervention) completing the month 6 assessment; those who completed the assessment did not differ from those who did not complete the assessment [19]. While participants across the two groups had comparable levels of knowledge, advocacy, stigma, and CC-management self-efficacy (see Table 1), the alters of index participants in the intervention group reported significantly increased CC prevention advocacy at month 6, compared to those in the control group, as measured by each item of the advocacy scale as well as the mean item score (see Table 2).

**Table 1:** Baseline comparisons of the CC prevention advocacy measure (individual items and scale score), along with the potential mediators across the alters of index participants in the control (*n* = 45) and intervention (*n* = 58) groups

**Measures**	**Control**	**Intervention**	***p*-value**
*CC prevention advocacy items*			
Discussed the importance of getting screened for CC risk *(scale: 1–5)*	2.02 (0.58)	2.19 (0.85)	0.260
Discussed how and/or where to get screened for CC risk *(scale: 1–5)*	2 (0.60)	2.14 (0.93)	0.390
Discussed how to prevent CC *(scale: 1–5)*	1.27 (0.45)	1.34 (0.61)	0.470
Discussed the need to use condoms to limit the risk of getting HPV-infected and CC *(scale: 1–5)*	1.11 (0.32)	1.15 (0.52)	0.620
Discussed the benefits of having fewer sexual partners to limit the risk of getting HPV-infected and CC *(scale: 1–5)*	1.13 (0.34)	1.22 (0.53)	0.320
Discussed the importance of getting treatment, if screening shows signs of CC risk *(scale: 1–5)*	1.84 (0.60)	1.81 (0.74)	0.800
Mean CC prevention advocacy score *(scale: 1–5)*	1.56 (0.37)	1.64 (0.58)	0.420
*Mediators*			
CC knowledge *(scale: 0–16)*	4.76 (2.60)	5.57 (2.83)	0.140
CC enacted stigma *(scale: 1–3)*	1.57 (0.42)	1.58 (0.39)	0.950
Level of CC prevention advocacy received by alter from index *(scale: 0–3)*	2 (0.1)	2.02 (0.08)	0.026***
CC risk management self-efficacy *(scale: 0–10)*	6.95 (1.56)	7.37 (1.40)	0.154
Observations	45	58	

* *p* < 0.1, ** *p* < 0.05, *** *p* < 0.01

**Table 2:** Intervention effects on month 6 measures of alter cervical cancer (CC) prevention advocacy (full scale and each item) and potential mediators

	Outcome measures [beta (SE)]^a^							Potential mediators [beta (SE)]^a ^			
	(1) Discussed the importance of getting screened for CC risk (scale: 1–5)	(2) Discussed how and/or where to get screened for CC risk (scale: 1–5)	(3) Discussed how to prevent CC (scale: 1–5)	(4) Discussed the need to use condoms to limit the risk of getting HPV-infected and CC (scale: 1–5)	(5) Discussed the benefits of having fewer sexual partners to limit the risk of getting HPV-infected and CC (scale: 1–5)	(6) Discussed the importance of getting treatment, if screening shows signs of CC risk (scale: 1–5)	(7) Mean prevention advocacy by alter (scale: 1–5)	(8) CC knowledge (scale: 0–16)	(9) CC enacted stigma (scale: 1–3)	(10) Level of CC prevention advocacy received by alter from index (scale: 0–3)	(11) CC risk management self-efficacy (scale: 0–10)
Treatment (binary)	1.56*** (0.24)	1.61*** (0.26)	1.82*** (0.26)	1.74*** (0.26)	1.76*** (0.25)	1.91*** (0.22)	1.74*** (0.23)	4.69*** (0.65)	0.07 (0.08)	0.35*** (0.09)	2.70*** (0.72)
Observations	103	103	103	103	103	103	103	103	103	103	103
Reference (control) mean at month 6	2.2 (0.89)	2.02 (0.99)	1.42 (0.75)	1.24 (0.71)	1.2 (0.69)	2.02 (0.87)	1.68 (0.69)	6.13 (2.93)	1.6 (0.36)	1.91 (0.48)	6.58 (2.11)
Socio-demographic controls^b^	X	X	X	X	X	X	X	X	X	X	X

* *p* < 0.1, ** *p* < 0.05, *** *p* < 0.01

^a^The beta [SE] values were estimated using multiple linear regression models, with the month 6 measure as the outcome and the intervention assignment as the independent variable

^b^The regressions controlled for the baseline measure, alter age, secondary education status, and the presence of a main sexual partner

### Mediation of the Intervention Effect on Alter CC Prevention Advocacy

Table 2 also shows the results of the regression models evaluating the effect of the intervention on the list of potential mediators from the pilot trial data. Alters of women in the intervention group reported a significantly greater increase in CC-related knowledge, CC risk management self-efficacy, and receipt of CC screening advocacy from their referring index participant at month 6, compared with the alters in the control group. All three of the above measures at month 6 were found to be significantly correlated with alter engagement in CC prevention advocacy at month 6 (CC knowledge: *r* = 0.79, *p* < 0.001; level of CC prevention advocacy received by alter from index: *r* = 0.46, *p* < 0.001; CC risk management self-efficacy: *r* = 0.39, *p* < 0.001).

With statistically significant indirect [beta (bootstrap 95% CI) = 1.03 (0.71–1.44)] and direct [beta (bootstrap 95% CI) = 0.61 (0.20–1.06)] effects, change in alter CC knowledge partially mediated the effects of the intervention on alter CC prevention advocacy (see Table 3). CC risk management self-efficacy and receipt of CC screening advocacy from the referring index participant did not mediate the intervention effects on CC prevention advocacy by alters.

**Table 3:** Regression models examining potential mediators of intervention effect on alter cervical cancer (CC) prevention advocacy at month 6

	**Alter CC-related knowledge**		**Alter CC risk management self-efficacy**		**Level of CC prevention advocacy that alter received from index participant**	
	Without mediator	With mediator	Without mediator	With mediator	Without mediator	With mediator
	*Beta (SE); p*	*Beta (SE); p*	*Beta (SE); p*	*Beta (SE); p*	*Beta (SE); p*	*Beta (SE); p*
Intervention	1.64 (0.22); < 0.001	0.61 (0.21); 0.004	1.69 (0.22); < 0.001	1.45 (0.26); < 0.001	1.67 (0.23); < 0.001	1.49 (0.25); < 0.001
Alter CC knowledge (baseline)	0.14 (0.04); < 0.001	0.06 (0.04); 0.115				
Alter CC knowledge (month 6)		0.22 (0.03); < 0.001				
Alter CC risk management self-efficacy (baseline)			0.18 (0.1); 0.073	0.15 (0.09); 0.106		
Alter CC risk management self-efficacy (month 6)				0.09 (0.05); 0.075		
Level of CC prevention advocacy received by alters (baseline)					1.69 (1.87); 0.369	1.49 (1.75); 0.398
Level of CC prevention advocacy received by alters (month 6)						0.54 (0.26); 0.043
Direct effect [*beta (bootstrap 95% CI)*]	0.61 (0.20– 1.06); *p* = 0.005		1.45 (0.83–2.06); *p* < 0.001		1.49 (0.96–1.96); *p* < 0.001	
Indirect effect [*beta (bootstrap 95% CI)*]	1.03 (0.71–1.44); *p* < 0.001		0.24 (0.14–0.81); *p* = 0.280		0.19 (− 0.02–0.41); *p* = 0.056	

The beta coefficient and standard error (SE) values were estimated using multiple linear regression models and included alter demographics (age, secondary education status, and relationship status) as covariates

### Moderators of the Intervention Effect on Alter CC Prevention Advocacy

No variables were found to significantly moderate the intervention effect on the alter measure CC prevention advocacy (data not shown).

## Discussion

Prior analysis of the pilot trial data showed that the GC-CCP intervention not only had an effect on increased CC prevention advocacy among index participants who received the intervention, but also increased advocacy among the alters of index participants in the intervention group [19]. This finding suggests that this network-driven approach to increasing CC screening uptake may result in a diffusion of increased CC prevention advocacy throughout social networks. The analysis reported here sought to examine the mechanisms that drive this intervention effect on increased engagement in CC prevention advocacy among alters, and the type of alters for whom the intervention had the greatest impact in terms of increased advocacy. We found that the intervention effect on increased CC prevention advocacy by alters was partially mediated by an increase in their knowledge about CC and that this intervention effect may generalize across a range of values among the measured alter characteristics. These results make us cautiously optimistic about the role of knowledge pertaining to CC driving the intervention effects on alter engagement in CC prevention advocacy, which may be tested further in a future, fully-powered trial of GC-CCP.

Our conceptual framework suggested several cognitive processes that may mediate the effect of the GC-CCP intervention on increased CC prevention advocacy among the alters targeted with advocacy by the index participants, in addition to the level of advocacy received by the alters (see Fig. 1). We expected that alters receiving prevention advocacy (as with those who reported getting screening for CC [30]) would exhibit an increase in their knowledge, a decrease in enacted CC stigma, and an increase in their self-efficacy for CC risk management. With the exception of CC stigma, the intervention had the expected effect on each of these constructs, as well as increased receipt of CC screening advocacy from the referring index participant. However, only CC-related knowledge partially mediated the intervention effect on alter CC prevention advocacy. Knowledge about CC, including the nature of the disease, how to prevent it, and the available treatments, may contribute to confidence and motivation to start conversations about CC and the importance of screening. It is worth noting that prior analysis revealed that the intervention effect on increased CC prevention advocacy among index participants was fully mediated by increased CC-related knowledge [31], further highlighting the importance of educating women about the facts and myths of CC as part of the intervention advocacy training.

It was surprising to note that none of the other variables mediated the intervention effects on alter CC prevention advocacy. The prevailing low levels of enacted stigma in our sample, coupled with the small sample size, may have impeded our ability to assess this as a mediator. It is possible that CC risk management self-efficacy is not a direct mediator of the intervention effects on alter CC prevention advocacy but rather is a consequence of increased CC knowledge, which in turn increases risk management self-efficacy. Lastly, our conceptual model stipulates advocacy self-efficacy to be a potential mediator of the intervention effect on increased advocacy, but we did not assess this variable among alters (this construct was only measured among index participants). Future research with a larger sample and more complete assessment battery will enable further evaluation of the mediators of the intervention effect on advocacy.

The moderator analysis was carried out to identify alter characteristics for whom the pilot intervention may have had a greater impact in increasing alter CC prevention advocacy. Our inability to identify any moderators of the intervention effect suggests that intervention effect on alter advocacy may be generalizable across a range of ages, education levels, relationship status, and social support. This finding differs from those pertaining to similar analyses carried out for index participants enrolled in the pilot trial [31], where we found that the intervention effects on increased advocacy were stronger for women with no secondary education, and who had a main sex partner. The negative finding regarding social support was surprising, considering our expectation that women who were well supported within their social networks might be more inclined, and more confident to initiate conversations around CC screening. The alter survey, which was shorter than that of the index participants, did not include a number of factors, such as how well connected the alters were with the women in their social networks, how much they trusted the women in their social networks, and how strongly positioned they were within their social networks to be able to carry out meaningful conversations about CC. These factors may influence the willingness to initiate conversations and to share experiences about CC with those within one’s social network. Several of these factors were found to moderate the effect of the intervention on increased advocacy among the index participants.

Limitations to our analysis include the selection bias that may be in our sample, as the alters were among the social network members who the index participants had shared their CC screening experience with and were comfortable referring to the study. Importantly, validated measures or measures used by other research groups were not available for most constructs, resulting in the need for our team to develop many of the measures used. Internal reliability statistics were moderate or good for most of these measures, but further psychometric evaluation is needed in future studies. Other limitations include the small sample size (given the pilot nature of the study), and limited statistical power, although many statistically significant associations were identified. As such, future work that tests the effectiveness of the intervention in the population may address many of these limitations and provide stronger evidence pertaining to the mechanisms that drive the intervention effect on alter engagement in prevention advocacy.

Our findings highlight the diffusion of advocacy that resulted from the GC-CCP advocacy training intervention and the importance of the intervention to include education about CC-related facts and myths. CC-related knowledge served to influence the intervention’s effect on increased CC prevention advocacy at both the first-degree level (i.e., by index participants to their alters, as previously reported) and second-degree level (i.e., by alters to their social network members). Network-based interventions such as GC-CCP have the potential to diffuse CC-related knowledge, alleviate CC stigma, and improve motivation for CC screening, via engagement in CC prevention advocacy, thereby providing a vital contribution to CC prevention and control. The intervention could additionally be applied to other settings outside of Uganda, particularly in areas with strong community-based networks.

## Supplementary Information

Below is the link to the electronic supplementary material.

Supplementary file1 (DOCX 15.8 KB)

## Data Availability

De-identified dataset and statistical code are available to researchers upon submission of proposal and review by the study team.

## Abbreviations

CC: Cervical cancer
RCT: Randomized controlled trial
UCI: Uganda Cancer Institute
RHHJ: Rays of Hope Hospice Jinja
GC-CCP: Game Changers for Cervical Cancer Prevention
CI: Confidence intervals
