# Supplementary material for: Increased Knowledge Mediates the Effect of Game Changers for Cervical Cancer Prevention on Diffusion of Cervical Cancer Screening Advocacy Among Social Network Members in a Pilot Trial
Source: Int J Behav Med. Author manuscript; Available in PMC 2025 Oct 1. (PMC10904666; doi:10.1007/s12529-023-10217-7)
Supplement: CC knowledge scale [file NIHMS1942170-supplement-CC_knowledge_scale.docx]

**Supplementary File 2**

**Cervical Cancer Knowledge Scale**

| **The next questions ask about your knowledge of cervical cancer, including risk factors, screening and treatment for cervical cancer.** | |
| --- | --- |
| alt_B1: Cervical cancer is caused by a virus called Human Papilloma Virus (HPV). | [1] True  [2] False  [3] Don’t know  [99] N/A or refuses |
| alt_B2: In Uganda, what is the most common way in which people contract human papilloma virus (HPV)? | [1] Sexual intercourse without condoms  [2] Sharing of utensils or cups  [3] Kissing  [4] All of the above  [5] Don’t know  [99] N/A or refuses |
| alt_B3: There is a vaccine that can prevent HPV infection. | [1] True  [2] False  [3] Don’t know  [99] N/A or refuses |
| alt_B4: Cervical cancer is the most common form of cancer among Ugandan women | [1] True  [2] False  [3] Don’t know  [99] N/A or refuses |
| alt_B5: Men can be infected with HPV | [1] True  [2] False  [3] Don’t know  [99] N/A or refuses |
| alt_B6: After how long does HPV infection usually show signs of risk for cervical cancer? | [1] 2-3 years  [2] 10-20 years  [3] 6 months  [4] Don’t know  [99] N/A or refuses |
| alt_B7: Being HIV+ increases a woman’s chances of getting cervical cancer? | [1] True  [2] False  [3] Don’t know  [99] N/A or refuses |
| alt_B8: Treatment for pre-cancerous lesions can prevent cervical cancer | [1] True  [2] False  [3] Don’t know  [99] N/A or refuses |
| alt_B9: A biopsy is used to determine whether cervical cancer is present. | [1] True  [2] False  [3] Don’t know  [99] N/A or refuses |
| alt_B10: Women who are **not** HIV-infected should be screened how often for cervical cancer? | [1] Every year  [2] Every 5 years  [3] Every 3 years  [4] Don’t know  [99] N/A or refuses |
| alt_B11: Women who are HIV-infected should be screened how often for cervical cancer? | [1] Every year  [2] Every 5 years  [3] Every 3 years  [4] Don’t know  [99] N/A or refuses |
| alt_B12: When should women first be screened for cervical cancer? | [1] When she starts to notice symptoms that are common with cervical cancer  [2] Soon after she first becomes sexually active  [3] After menopause  [4] Don’t know  [99] N/A or refuses |
| alt_B13: How long should a woman wait to have sexual intercourse after receiving treatment for lesions? | [1] Six days  [2] Six weeks  [3] Six months  [4] Don’t know  [99] N/A or refuses |
| alt_B14: Which of the following could be signs of cervical cancer? | [1] Vaginal bleeding between menstrual periods OR after menopause [Menopause is when a woman’s periods have stopped permanently]  [2] A persistent smelly vaginal discharge  [3] Discomfort, pain or bleeding during and after sex  [4] Persistent lower abdominal/pelvic pain  [5] All of the above  [6] Don’t know  [99] N/A or refuses |
| alt_B15: The screening procedure for cervical cancer can damage the uterus. | [1] True  [2] False  [3] Don’t know  [99] N/A or refuses |
| alt_B16: After having treatment in the uterus for any sign of cervical cancer risk, a woman may no longer be able to conceive a child | [1] True  [2] False  [3] Don’t know  [99] N/A or refuses |
